# The Polyhedral Matrix Configuration (PMC) Technique: A Retrospective Cohort Study of Geometric Standardization of Acellular Dermal Matrix Wrapping and Operative Efficiency in Prepectoral Breast Reconstruction

**DOI:** 10.3390/jcm15031226

**Published:** 2026-02-04

**Authors:** Hyung-suk Yi, Jeong-jin Park, Jin-hyung Park, Yoon-soo Kim

**Affiliations:** Department of Plastic and Reconstructive Surgery, Kosin University Gospel Hospital, Kosin University College of Medicine, Busan 49267, Republic of Korea; sencha21@naver.com (H.-s.Y.); jayjay03157@gmail.com (J.-j.P.); atreyue@naver.com (J.-h.P.)

**Keywords:** breast cancer, breast reconstruction, mammaplasty, mastectomy, acellular dermal matrix

## Abstract

**Background**: Prepectoral breast reconstruction with an acellular dermal matrix (ADM) typically requires intraoperative manual tailoring, introducing structural variability and workflow delays. We developed the Polyhedral Matrix Configuration (PMC) technique—a geometric method for standardizing ADM shell creation—and compared it to our traditional “tear-drop” wrap to determine whether standardization improves structural integrity and operative efficiency. **Methods**: We reviewed all consecutive 227 patients undergoing immediate prepectoral reconstruction from January 2021 to December 2024 (tear-drop group: n = 155; PMC group: n = 72). PMC transforms planar ADM into a 3D dome using pre-designed wedge resections and butt-joint sutures, eliminating material overlap. Standardization permits back-table fabrication during mastectomy (“parallel two-team workflow”). We excluded bilateral cases for consistent operative time assessment and performed subgroup analysis to control for higher robotic mastectomy rates in the PMC cohort. **Results**: PMC reduced the plastic surgery time by a mean of 44.6 min (95% CI: 35.2–54.0) (*p* < 0.001), with subgroup analysis confirming efficiency gains across both conventional (32.8 min, 95% CI: 20.1–45.5, *p* < 0.001) and robotic mastectomies (60.8 min, 95% CI: 47.3–74.3, *p* < 0.001). Despite zero-overlap design, PMC showed no increase in major complications (*p* > 0.99) and lower rates of visible rippling (odds ratio 0.28, 95% CI: 0.08–0.97, *p* = 0.032). BREAST-Q “Satisfaction with Breasts” scores were higher in the PMC group (mean difference +7.3 points, 95% CI: 3.1–11.5, *p* = 0.001). **Conclusions**: Geometric standardization enables both design precision and operative efficiency. By separating reconstruction preparation from mastectomy through a reproducible protocol, PMC reduces the operative time while improving aesthetics through stable, single-layer construction.

## 1. Introduction

Prepectoral prosthetic breast reconstruction has gained favor over subpectoral techniques, avoiding donor-site morbidity and eliminating animation deformity [1,2]. The acellular dermal matrix (ADM) serves as the structural interface between the implant and mastectomy skin flap [3,4,5]. Its proper application significantly impacts both aesthetic outcomes and complication rates. Recent meta-analyses have demonstrated that ADM coverage reduces visible rippling rates from approximately 15–25% to 4–8% in prepectoral reconstruction [6,7]. Furthermore, adequate ADM wrapping has been associated with lower capsular contracture rates (Baker III/IV: 3–7% with ADM vs. 12–18% without ADM) [8]. Patient-reported outcomes similarly reflect these benefits, with BREAST-Q ‘Satisfaction with Breasts’ scores averaging 70–80 points in ADM-assisted prepectoral series compared to 60–68 points in non-ADM techniques [9,10].

Traditional prepectoral methods, such as our ‘tear-drop’ wrap [11], rely on intraoperative manual tailoring. In this technique, a large ADM sheet is wrapped around the implant with edges overlapping at the posterior and superior aspects to create a tear-drop shape. However, this manual molding creates overlapping materials that lead to structural inconsistency. Traditional prepectoral methods, such as our “tear-drop” wrap [11], rely on intraoperative tailoring. Surgeons manually mold a flat ADM over a spherical implant within the surgical field. This approach has two inherent limitations: overlapping materials create structural inconsistency, and sequential processing reduces workflow efficiency In our tear-drop series (Kim et al. 2022) [11], the mean plastic surgery reconstruction time was 143.5 ± 42.9 min, reflecting the time required for surgeons to manually mold a flat ADM [12,13].

The Polyhedral Matrix Configuration (PMC) technique addresses these challenges. Based on “Surgical Origami” principles [13,14], it applies a geometric algorithm to convert 2D sheets into 3D volumes without overlap. The PMC technique employs a mathematically fixed 1:2 base-to-height ratio for wedge resections, derived from polyhedral geometry principles. Unlike freehand tailoring, where each surgeon estimates proportions individually, this ratio is objectively determined and eliminates subjective judgment in template creation. The ability to fabricate the PMC shell on a back table during mastectomy—completely separate from the patient-specific chest wall anatomy—demonstrates that the construct’s standardization is sufficient for it to be created independently. This is a calculation-driven approach that maximizes reproducibility [15,16]. This study demonstrates that PMC serves dual functions: enhancing architectural stability via zero-overlap butt-joints, and reducing the operative time through a parallel two-team workflow enabled by geometric standardization [17,18].

## 2. Materials and Methods

### 2.1. Study Design and Patient Selection

We reviewed all consecutive 227 patients who underwent immediate prepectoral implant-based reconstruction from January 2021 to December 2024. Patients were divided into tear-drop (n = 155, historical control) and PMC (n = 72) groups. All underwent nipple-sparing mastectomy with immediate direct-to-implant reconstruction. Only unilateral cases were analyzed to ensure a consistent operative efficiency comparison and eliminate confounding from prolonged anesthesia or concurrent procedures.

### 2.2. Ethical Considerations

All study participants provided written informed consent for database storage and research use. The study protocol was approved by the Institutional Review Board of Kosin University Gospel Hospital of Korea (KUGH 2025-06-007). All procedures were performed in accordance with the ethical standards of the Institutional and National Research Committee and the 1964 Declaration of Helsinki and its later amendments.

### 2.3. The PMC Design: “Surgical Origami” Principles

**Figure 1:**
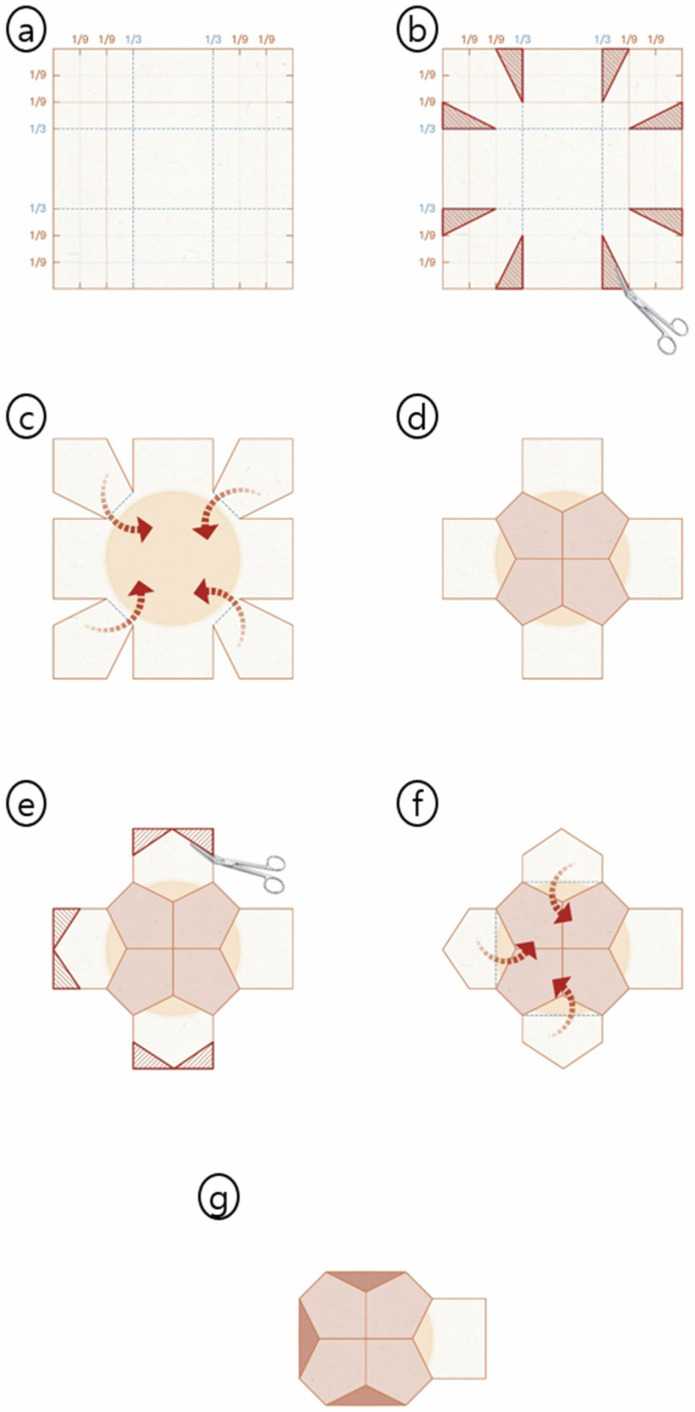
PMC technique assembly protocol. (**a**) Template marking with 9 × 9 grid. (**b**) Wedge resection of triangular sections at 1:2 base-to-height ratio. (**c**) Corner flaps fold to form octagonal base. (**d**) Butt-joint sutures create zero-overlap alignment. (**e**) Peripheral trimming shapes sidewall contours. (**f**) Completed 3D shell with anterior access window. (**g**) Final construct provides tension-free coverage.

PMC uses a predefined geometric template to transform a 2D planar ADM into a 3D polyhedral shell without material overlap (Figure 1). Unlike freehand tailoring in the tear-drop method, PMC follows mathematically derived origami-based patterns. ADM edges are approximated using the butt-joint technique, eliminating bulk from overlapping layers. Seams are secured with 3-0 absorbable monofilament in an interrupted interlocking fashion, distributing tension evenly to maintain the 3D volume under the implant weight.

### 2.4. Detailed Surgical Technique

The framework shown in Figure 1 is executed through a standardized back-table protocol during mastectomy.

**Figure 2:**
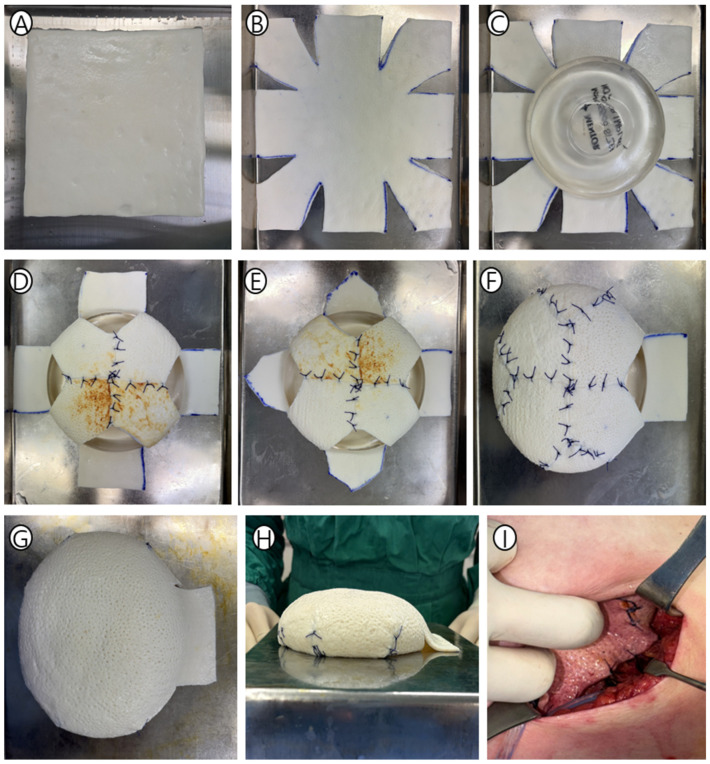
PMC shell assembly during surgery. (**A**) ADM sheet secured to sterile sizer on back table. (**B**–**E**) Sequential wedge resection and butt-joint suturing create 3D volume. (**F**,**G**) Completed dome-shaped PMC module ready for insertion. (**H**) Final view after implant insertion. The zero-overlap construct creates a smooth interface with overlying tissue. (**I**) The window’s edge anchors to pectoralis major fascia.

Step 1: ADM Selection and 9 × 9 Grid Marking (Figure 1a and Figure 2A). ADM is selected based on the anticipated implant volume, with adjustment for chest wall dimensions and skin envelope quality. We utilized Bellacell (HansBiomed, Seoul, Republic of Korea), Megaderm (L&C BIO, Seoul, Republic of Korea), and CGDerm Onestep (CG BIO, Seongnam, Republic of Korea) acellular dermal matrices. For the case in Figure 2, we used an 18 × 18 cm ADM for a 275 cc implant. The ADM is placed flat on a sterile tray, and its surface is marked with a 9 × 9 grid, creating reference points at 2/9, 1/3, 2/3, and 7/9 positions along both axes.

Step 2: Corner Resection—The 1:2 Ratio (Figure 1b and Figure 2B). Eight triangular wedges are excised at the four corners with a critical 1:2 base-to-height ratio. This ratio provides the optimal folding angle for eight peripheral segments to meet centrally without tension. Excised triangular fragments are saved for potential intraoperative adjustment.

Step 3: Zero-Overlap Alignment—Primary Coverage (Figure 1c and Figure 2C,D). A sterile breast sizer matching the intended final implant volume is placed centrally on the prepared template. Four corner flaps fold inward and are approximated with interrupted absorbable sutures Vicryl 3-0 (Ethicon, Raritan, NJ, USA), creating an octagonal base. This distributes forces across eight anchor points, providing uniform support.

Step 4: Peripheral Trimming and Sidewall Formation (Figure 1d,e and Figure 2E–G). Peripheral edges are trimmed to create tapered contours for continuous sidewalls. ADM segments are elevated and sutured edge-to-edge in a butt-joint fashion. The anterior access window serves three purposes: entry for implant insertion, anchor to pectoralis major fascia, and soft upper pole transition.

**Figure 3:**
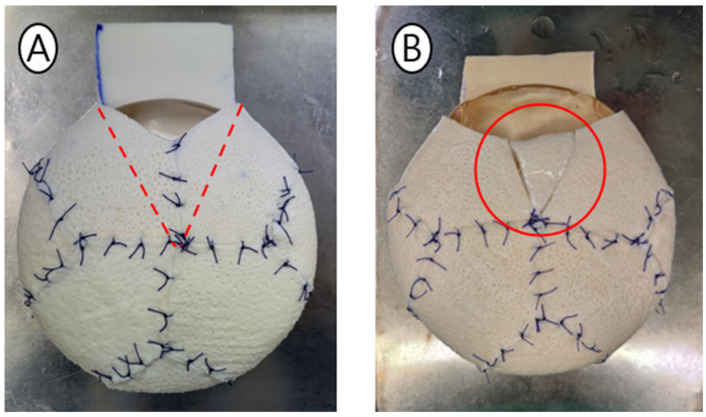
Intraoperative customization options. (**A**) Volume Reduction: For smaller final implants, an ADM wedge is excised to tighten the shell. (**B**) Volume Expansion: For larger implants, the suture line is released and a preserved triangular patch is inserted to expand the envelope.

Step 5: Final Shell Configuration (Figure 3). Despite the standardized template, intraoperative variability is accommodated through minor adjustments. For smaller final implants, a small ADM wedge can be excised to tighten the shell. For larger implants, preserved triangular patches can be inserted to expand the envelope.

Step 6: Implant Insertion and Fixation (Figure 2H,I). Once mastectomy is complete and hemostasis achieved, the prefabricated PMC shell is transferred to the surgical field. The sizer is removed through the anterior window, and the final breast implant is inserted. The window’s superior edge anchors to pectoralis major fascia at 12 o’clock or, in cases with upper pole depression deformity, positioned to fill the axillary hollow. A closed suction drain is placed subcutaneously, and mastectomy flaps are closed in a standard fashion.

### 2.5. Operative Workflow: Design-Enabled Parallelization

**Figure 4:**
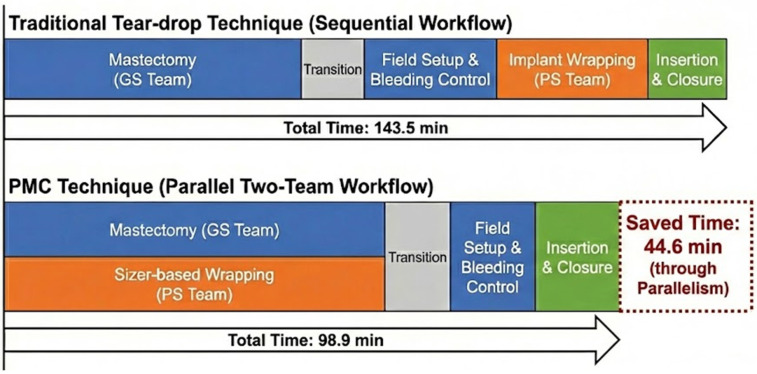
Operative workflow comparison. (**Top**): Traditional tear-drop technique—reconstruction starts after mastectomy completion. (**Bottom**): PMC technique—plastic surgery team fabricates ADM shell on back table during mastectomy. Red dashed box indicates mean time saved (44.6 min) through parallel processing.

**Figure 5:**
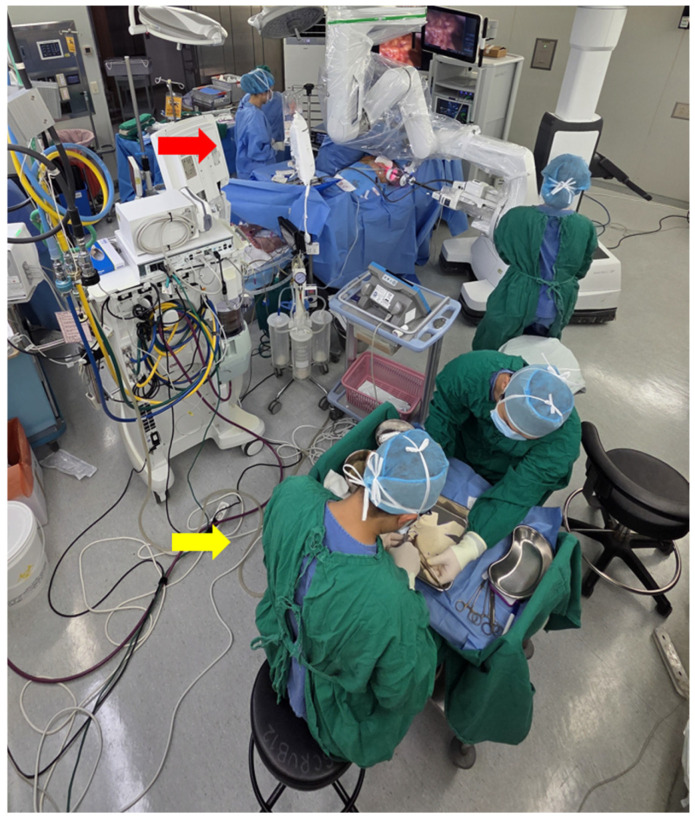
Parallel two-team workflow in practice. Red arrow: General surgery team performs nipple-sparing mastectomy. Yellow arrow: Plastic surgery team simultaneously fabricates PMC ADM shell on sterile back table. This concurrent approach readies reconstruction material by mastectomy completion.

A key distinction in this study is workflow modification enabled by PMC design (Figure 4). Traditional methods (tear-drop group) require ADM tailoring only after mastectomy completion and measurements. Because PMC design standardizes on the implant size rather than variable chest wall contours, the ADM shell could be fabricated on a sterile back table during mastectomy (Figure 5)—a “decoupled workflow” dependent on PMC design reproducibility.

### 2.6. Statistical Analysis

All continuous variables were assessed for normality using the Kolmogorov–Smirnov test prior to comparative analysis. Variables demonstrating a normal distribution (age, BMI, operative time, implant volume, BREAST-Q scores) were analyzed using Student’s *t*-test. Variables not meeting normality assumptions were analyzed using the Mann–Whitney U test. In our dataset, all primary and secondary outcome measures demonstrated a normal distribution, and therefore, parametric testing was appropriate throughout.

Continuous variables appear as means ± standard deviations with 95% confidence intervals where applicable and were compared with Student’s *t*-test or the Mann–Whitney U test depending on the distribution. Categorical variables were analyzed using the chi-square or Fisher’s exact test. The plastic surgery operative time was the pre-specified primary endpoint. Secondary endpoints included complication rates, aesthetic outcomes (AIS), and patient-reported outcomes (BREAST-Q). Subgroup analyses and individual BREAST-Q domain comparisons should be interpreted as hypothesis-generating exploratory endpoints. *p*-values < 0.05 were significant. SPSS 25.0 (IBM Corp., Armonk, NY, USA) was used for analysis.

Definition of Plastic Surgery Time:

- Start point: Completion of mastectomy and hemostasis, marked by the general surgery team stepping back from the operative field.
- End point: Completion of skin closure.
- Data source: Prospectively recorded from electronic anesthesia records, with times documented by circulating nurses at each phase transition.
- Included phases: All reconstructive procedures, including ADM positioning, implant insertion, fixation suturing, drain placement, and wound closure.

Critical distinction for PMC workflow: In the PMC group, back-table ADM shell fabrication occurs concurrently during the mastectomy phase and is therefore not included in the sequential plastic surgery time. This parallel processing is the structural basis for operative efficiency.

Aesthetic Assessment Protocol: Standardized clinical photographs were obtained at 6-month follow-up using identical camera settings (Canon EOS 800D (Canon Inc., Tokyo, Japan), 50 mm lens), fixed distance (1.5 m), and standardized studio lighting. Patients were positioned in the same frontal, lateral, and oblique views according to our institutional photography protocol. The five board-certified plastic surgeons who performed blinded AIS assessments demonstrated excellent agreement, with an intraclass correlation coefficient (ICC) of 0.87 (95% CI: 0.82–0.91).

Generative AI tools (e.g., ChatGPT-5, OpenAI, San Francisco, CA, USA) were used solely for English language editing and polishing to improve readability. The authors verify that no AI tools were used in the generation of scientific data or figures.

## 3. Results

### 3.1. Patient Demographics

Table 1 shows the baseline characteristics. The mean age was comparable between groups (49.5 ± 8.2 vs. 50.1 ± 7.9 years, *p* = 0.584). BMI averaged 23.8 vs. 23.5 kg/m^2^, *p* = 0.512. Robotic mastectomies were more common in the PMC group (68.1% vs. 37.4%, *p* < 0.001), prompting subgroup analysis.

### 3.2. Operative Time and Workflow Efficiency

PMC significantly reduced the plastic surgery operative time (Table 2). The mean plastic surgery time was 143.5 ± 42.9 min (Tear-drop) versus 98.9 ± 28.8 min (PMC), a 44.6 min reduction (95% CI: 35.2–54.0, *p* < 0.001). Subgroup analysis confirmed efficiency gains: in the robotic mastectomy subgroup, the plastic surgery time decreased from 158.4 to 97.6 min (60.8 min saved, 95% CI: 47.3–74.3, *p* < 0.001); for conventional mastectomy, the plastic surgery time decreased from 134.6 to 101.8 min (32.8 min saved, 95% CI: 20.1–45.5, *p* < 0.001). This workflow efficiency persisted across both mastectomy types despite higher robotic rates in the PMC cohort.

Postoperative recovery metrics showed no significant differences between groups (Table 2), with a comparable hospital length of stay (14.3 ± 3.8 vs. 14.1 ± 3.5 days, *p* = 0.684) and drain removal time (13.5 ± 3.6 vs. 13.2 ± 3.3 days, *p* = 0.541). This indicates that the operative efficiency of PMC was achieved without negatively impacting the recovery time.

### 3.3. Safety and Aesthetic Outcomes

Major complications requiring reoperation showed no significant difference between groups (2.6% tear drop vs. 2.8% PMC, *p* > 0.99, Table 3). Infection, seroma, and implant loss were comparable. Visible rippling was significantly reduced with PMC (4.2% vs. 13.5%, OR 0.28, 95% CI: 0.08–0.97, *p* = 0.032), with BREAST-Q “Satisfaction with Breasts” scores significantly higher in the PMC group (79.8 vs. 72.5, mean difference +7.3, 95% CI: 3.1–11.5, *p* = 0.001, Table 4). Physical well-being (Chest) also improved (90.4 vs. 86.9, mean difference +3.5, 95% CI: 0.8–6.2, *p* = 0.012).

BREAST-Q response rates were 88.4% (137/155) in the tear-drop group and 87.5% (63/72) in the PMC group, with no significant difference between groups (*p* = 0.85). Complete case analysis was performed for BREAST-Q outcomes. Patients with missing data were excluded only from PRO analyses; baseline characteristics of non-responders did not differ significantly from responders in either group.

Blinded aesthetic evaluation using the Aesthetic Item Scale confirmed superior outcomes in the PMC group (Table 5), with significant improvements in “Breast Shape Naturalness” (4.21 vs. 3.86, mean difference +0.35, 95% CI: 0.14–0.56, *p* = 0.001) and “Contour Smoothness” (4.26 vs. 3.71, mean difference +0.55, 95% CI: 0.35–0.75, *p* < 0.001). The total AIS score was higher in the PMC (20.70 vs. 19.49, mean difference +1.21, 95% CI: 0.48–1.94, *p* = 0.001).

## 4. Discussion

### 4.1. Geometric Standardization and Operative Efficiency

This study demonstrates that geometric standardization in prepectoral breast reconstruction provides both structural benefits and operative efficiency. The 44.6 min reduction in operative time is our principal finding. One might argue that this efficiency stems solely from the “Two-Team Approach” rather than the design itself. We disagree. Geometric design is the prerequisite for a parallel workflow [17,18]. Without a standardized template, simultaneous back-table preparation remains impossible.

Traditional methods like our tear-drop wrap [11] require fitting the ADM to each patient’s chest wall defects in real time. PMC standardizes the ADM-to-implant relationship using a “Surgical Origami” template [13,14], separating reconstruction preparation from the mastectomy phase. This separation serves as the foundation for parallel processing [17,19].

Several lines of evidence support the reproducibility of the PMC technique. The PMC technique employs a mathematically fixed 1:2 base-to-height ratio for wedge resections, derived from polyhedral geometry principles. Unlike freehand tailoring, where each surgeon estimates proportions individually, this ratio is objectively determined and eliminates subjective judgment in template creation. Furthermore, the ability to fabricate the PMC shell on a back table during mastectomy—completely separate from the patient-specific chest wall anatomy—demonstrates that the construct’s standardization is sufficient for it to be created independently. Traditional techniques require real-time adaptation to each patient’s defect, inherently introducing variability. Supporting this interpretation, our subgroup analysis revealed that if operative time reduction were attributable solely to surgeon learning curves or team experience, we would expect variable effects depending on surgical complexity. Instead, PMC demonstrated statistically significant time savings in both conventional mastectomy (32.8 min, *p* < 0.001) and robotic mastectomy subgroups (60.8 min, *p* < 0.001). This consistency across different complexity levels suggests a systematic, technique-driven effect rather than operator-dependent improvement.

These workflow improvements align with the recent literature. Todd et al. [17] showed that systematic workflow modifications in autologous breast reconstruction produced progressive operative time reductions over 15 years. Canizares et al. [19] similarly reported that optimized team coordination significantly decreased the DIEP flap reconstruction duration. Our findings apply these principles to prepectoral implant-based reconstruction, demonstrating that geometric standardization enables workflow parallelization impossible with patient-specific tailoring.

### 4.2. Zero-Overlap Design: Structural and Aesthetic Outcomes

Several strategies have been proposed to minimize rippling in prepectoral reconstruction. Chopra et al. [20] highlighted the role of autologous fat grafting and strategic ADM reinforcement in reducing implant visibility. Longo et al. [21] described a hybrid dual-plane approach to mask the implant edge through partial muscle coverage. Unlike these methods, which often require additional donor sites or secondary procedures, the PMC technique utilizes a zero-overlap butt-joint design to create a stable, dome-shaped shell as a primary prevention strategy, maintaining a uniform thickness without adding bulk.

The PMC technique also addresses the broader question of ADM utility in prepectoral reconstruction. Recent multicenter studies have demonstrated that ADM-assisted prepectoral reconstruction yields lower complication rates than implant-only techniques, particularly in patients with thin mastectomy flaps [22]. Comparative analyses report reduced rippling (4–8% vs. 15–25%), lower capsular contracture (Baker III/IV: 3–7% vs. 12–18%), and improved implant position stability with ADM coverage [6,7,8]. BREAST-Q data from contemporary series demonstrate that ADM-assisted prepectoral reconstruction achieves “Satisfaction with Breasts” scores comparable to or exceeding autologous reconstruction (mean 70–82 points), attributed to smoother contours and reduced palpability [9,10].

Having established ADM’s value, we emphasize that how ADM is applied—specifically, single-layer zero-overlap construction versus multi-layer overlapping techniques—determines the final aesthetic outcome. This positions PMC as an optimization of an already beneficial material, not merely an alternative wrapping method.

Concerns about the mechanical stability of butt-joint constructs are valid in theory. However, they did not manifest clinically [23,24]. We observed no increase in seam failure or implant herniation. The multi-planar force distribution in the polyhedral shape likely accounts for this stability, mimicking the natural breast dome geometry more effectively than forced 2D wrapping [13,16].

The zero-overlap butt-joint design demonstrated clear clinical advantages. Rippling rates decreased from 13.5% to 4.2% (*p* = 0.032). This correlated with improved BREAST-Q satisfaction scores (79.8 vs. 72.5, *p* = 0.001) and superior Aesthetic Item Scale ratings for contour smoothness (4.26 vs. 3.71, *p* < 0.001). The structural refinement eliminates palpable ridges inherent to overlapping ADM layers [6,11,25]. This is particularly critical in patients with thin mastectomy skin flaps [3,26].

The stability observed despite zero-overlap construction supports the hypothesis that proper geometric design compensates for reduced material redundancy. Zhang et al. demonstrated that complete implant coverage with properly tensioned ADM effectively prevents capsular contracture even without overlapping layers [24]. Pittman et al. developed the P1 method to address upper pole rippling through strategic ADM placement, reporting similar improvements when overlapping materials were minimized [7].

### 4.3. Robotic Mastectomy and PMC Workflow

Robotic mastectomies were more common in the PMC group (68.1% vs. 37.4%, *p* < 0.001). Robotic procedures typically require a longer operative time [27,28]. Our subgroup analysis revealed that the PMC workflow saves sufficient reconstruction time to offset a longer robotic mastectomy duration. This finding suggests that PMC suits high-volume robotic centers particularly well, where minimizing the total anesthesia time is important for patient safety and turnover efficiency [15,29].

The combination of robotic surgery with prepectoral reconstruction represents a growing area of interest in breast surgery [30,31]. Roy et al. [28] noted that while robotic approaches increased operative times, they were associated with improved patient-reported outcomes. Khan et al. similarly reported decreased postoperative pain with robotic-assisted techniques [27]. Coupling robotic mastectomy with PMC’s efficient workflow may reduce time disadvantages while preserving robotic benefits. Vidya and Cawthorn [15] emphasized that future breast surgery advances will increasingly rely on reproducible, technology-enabled approaches that reduce surgeon-dependent variability.

### 4.4. Patient-Reported Outcomes

The higher BREAST-Q scores in the PMC group (79.8 vs. 72.5, *p* = 0.001) warrant discussion [9,10]. Objective aesthetic assessments using the Aesthetic Item Scale corroborated these subjective outcomes. Cohen et al. [10] noted that BREAST-Q satisfaction correlates strongly with the breast shape, symmetry, and absence of visible deformities. Our reduced rippling rates and enhanced architectural stability likely drove these improved outcomes. These scores exceed the mean satisfaction scores that Pusic et al. [9] reported (approximately 64 points).

**Figure 6:**
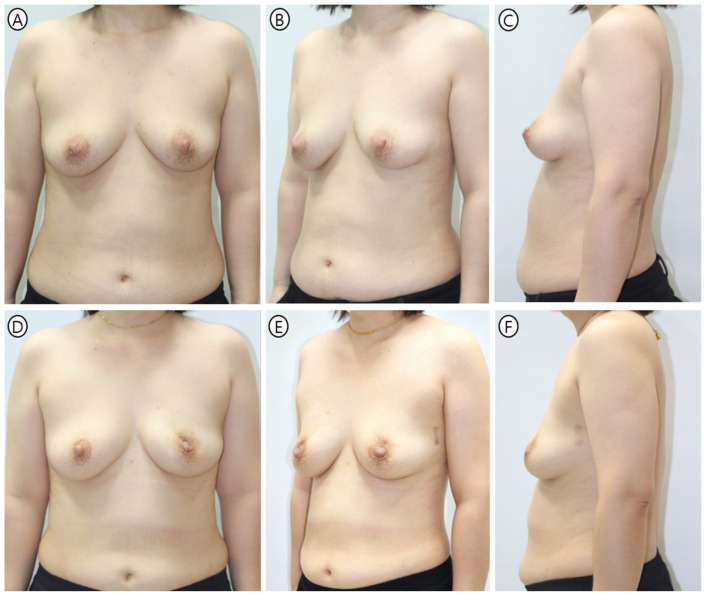
Representative case 1—preoperative and 6-month postoperative views. A 40-year-old patient (BMI 24.8 kg/m^2^) underwent immediate prepectoral reconstruction following left robotic nipple-sparing mastectomy. Reconstruction used a 235 cc Mentor smooth round implant wrapped in 18 × 18 cm^2^ Bellacell ADM via lateral vertical incision. (**A**–**C**) Preoperative baseline morphology. (**D**–**F**) Six-month postoperative results show maintained projection and symmetry with smooth upper pole transition despite thin skin envelope from robotic dissection.

**Figure 7:**
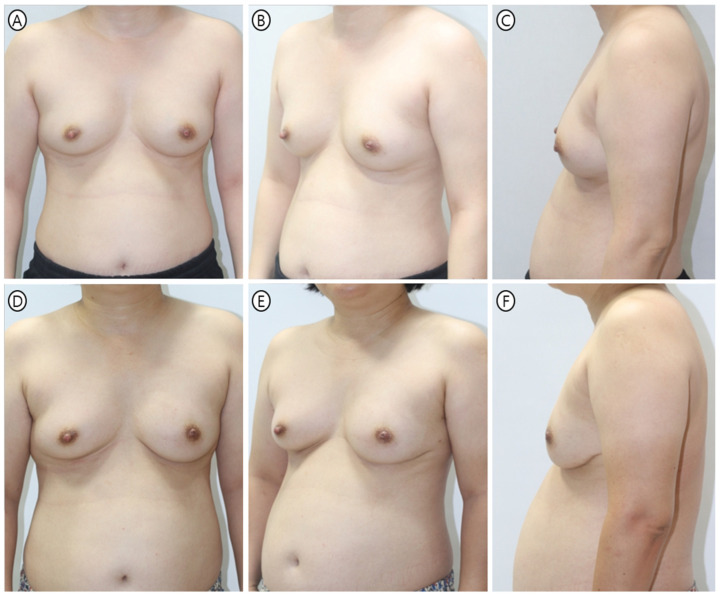
Representative case 2—PMC technique in conventional mastectomy. A 50-year-old female patient (BMI 24.7 kg/m^2^) underwent immediate prepectoral reconstruction following left conventional nipple-sparing mastectomy (specimen weight 249 g). A 215 cc Mentor MemoryGel smooth round implant (Classic profile; Mentor Worldwide LLC, Irvine, CA, USA) was wrapped in 18 × 18 cm Bellacell ADM (HansBiomed, Seoul, Republic of Korea) using PMC technique via inframammary fold incision. (**A**–**C**) Preoperative frontal, oblique, and lateral views showing moderately ptotic breast morphology with lower pole fullness. (**D**–**F**) Nine-month postoperative results demonstrate maintained projection and symmetry with smooth contour transition. No visible rippling or implant palpability occurred despite relatively lower BMI. The patient-reported BREAST-Q “Satisfaction with Breasts” score of 85 and Physical Well-being (Chest) score of 92 indicate high satisfaction with both aesthetic and functional outcomes.

Khan et al. reported similar BREAST-Q satisfaction scores (mean 82.6 points) in prepectoral reconstruction with anterior ADM coverage [32]. However, their study did not employ geometric standardization or parallel workflow optimization. Our technique’s efficiency gains therefore represent an additional advantage beyond aesthetic outcomes alone (Figure 6 and Figure 7).

### 4.5. Alternative Wrapping Techniques

While preformed ADM products such as Braxon^®^ (DEKA, Florence, Italy) offer convenience in some markets, they present two important limitations in our clinical context: (1) regulatory availability—preformed products are not currently distributed in Korea, limiting their practical application in our healthcare system; and (2) geometric flexibility—preformed shells come in fixed sizes, whereas PMC enables precise customization to individual implant dimensions using standard planar ADM sheets. This adaptability is particularly valuable in Asian populations where implant size distributions differ from Western cohorts. The PMC technique thus provides a universally applicable solution that leverages readily available materials while maintaining the standardization benefits typically associated with preformed products.

PMC shares conceptual ground with Hill and Buck’s “Butterfly Wrap” [12] regarding reproducibility and minimal ADM waste. However, the Butterfly Wrap focuses on tear-drop shapes for lower pole fullness, whereas PMC emphasizes a polyhedral geometry for uniform force distribution and complete coverage. Reitsamer and Peintinger [3] introduced complete porcine ADM coverage for prepectoral reconstruction with favorable early outcomes, but their technique relies more heavily on intraoperative customization rather than pre-templated geometric patterns. This potentially limits workflow optimization. The “double-crossed” ADM technique [23] involves multiple overlapping layers, potentially increasing material bulk and seroma risk [20]. Our zero-overlap design avoids these disadvantages while maintaining structural integrity.

### 4.6. Surgical Education and Future Applications

An often-overlooked advantage of geometric standardization is its impact on surgical education. A clear algorithmic template reduces the learning curve for prepectoral reconstruction. Standardized wrapping techniques benefit early-career surgeons by reducing anxiety about prosthesis wrapping and ADM sizing [12]. Tampaki and Tampakis [16] emphasized that standardization is necessary for proper scientific evaluation and cross-study comparison. PMC transforms reconstruction from an operator-dependent variable into a reproducible process.

PMC principles—geometric standardization and workflow optimization—align with current surgical innovation trends [15,18]. Technologies like 3D imaging and robotic assistance aim to reduce variability through systematic approaches [18,30]. Future PMC applications could incorporate 3D scanning to refine geometric templates based on the individual anatomy while maintaining standardization. Advanced imaging modalities such as indocyanine green angiography may further optimize intraoperative decisions regarding ADM placement and fixation [31].

### 4.7. Study Limitations

This study is limited by its retrospective nature and single-center design [33]. The use of a historical control design carries inherent limitations, including potential temporal and learning-curve biases related to the progressive evolution of surgical team experience and operating room organization [22]. However, several lines of evidence suggest our findings reflect true structural advantages of the PMC technique: (1) subgroup analysis demonstrated consistent efficiency gains across both mastectomy types, (2) all procedures were performed by the same surgical team at a single institution using consistent patient selection criteria, with all participating surgeons having completed more than 50 prepectoral reconstructions prior to the study period (representing a mature practice phase), and (3) the PMC technique introduces a fundamental workflow modification—parallel back-table fabrication during mastectomy—that is structurally distinct from iterative skill refinement.

Subgroup analysis mitigates selection bias regarding the mastectomy type, but prospective randomized controlled trials would provide higher-level evidence [34]. Long-term follow-up (>5 years) remains necessary to assess the butt-joint seam durability and long-term capsular contracture rates [8,35]. The exclusion of bilateral reconstruction cases, while necessary for an accurate operative time comparison, limits the generalizability to patients undergoing unilateral procedures. Learning curve effects may have influenced early PMC cases. Cost-effectiveness analysis would quantify the economic benefits of a reduced operative time.

Furthermore, while not observed in our study period (maximum 4-year follow-up), Breast Implant-Associated Anaplastic Large Cell Lymphoma (BIA-ALCL) remains a potential long-term risk in all implant-based reconstruction. We utilized smooth round implants in all cases, which are associated with a significantly lower risk profile compared to textured devices [36,37].

## 5. Conclusions

The PMC technique offers both aesthetic precision and operative efficiency through geometric standardization. By establishing a reproducible protocol, we have made prepectoral reconstruction more predictable and less operator-dependent. This approach reduces the operative time through workflow parallelization [17,19] and provides superior aesthetic outcomes by minimizing material overlap [6,7,25]. Our findings suggest that procedural workflow redesign, not just incremental technical refinements, can yield substantial improvements in both efficiency and outcomes.

## Figures and Tables

**Table 1:** Patient demographics and baseline characteristics.

Variable	Tear-Drop Group (n = 155)	PMC Group (n = 72)	*p*-Value
Age (years)	49.5 ± 8.2	50.1 ± 7.9	0.584
BMI (kg/m^2^)	23.8 ± 3.4	23.5 ± 3.1	0.512
Comorbidities			
Diabetes Mellitus	11 (7.1%)	5 (6.9%)	0.999 ^
Hypertension	19 (12.3%)	8 (11.1%)	0.805
Current Smoker	5 (3.2%)	2 (2.8%)	0.999 ^
Mastectomy Type			<0.001 *
Conventional (CNSM)	97 (62.6%)	23 (31.9%)	
Robot-Assisted (RANSM)	58 (37.4%)	49 (68.1%)	
Surgical Factors			
Specimen Weight (g)	315.2 ± 102.5	308.4 ± 98.1	0.645
Implant Volume (cc)	285.6 ± 85.4	295.2 ± 90.1	0.438
ADM Size (cm^2^)	324.8 ± 17.5	323.6 ± 16.2	0.624

Values are presented as mean ± SD or number (%). ^ Fisher’s exact test. * Statistically significant (*p* < 0.05), indicating higher proportion of robot-assisted surgeries in the PMC group.

**Table 2:** Operative efficiency stratified by mastectomy method.

Variable	Tear-Drop Group (n = 155)	PMC Group (n = 72)	95% CI	*p*-Value
Operative Time (min)				
Total Operative Time (Overall)	268.4 ± 58.2	252.6 ± 42.8	3.2–28.4	0.028 *
Plastic Surgery Time (Overall)	143.5 ± 42.9	98.9 ± 28.8	35.2–54.0	<0.001 **
PS Time in CNSM	134.6 ± 45.3	101.8 ± 22.7	20.1–45.5	<0.001 **
PS Time in RANSM	158.4 ± 33.9	97.6 ± 31.4	47.3–74.3	<0.001 **
Mean PS Time Saving (Adjusted)	Ref.	−44.6 min	35.2–54.0	<0.001 **
Recovery Metrics				
Hospital LOS (days)	14.3 ± 3.8	14.1 ± 3.5	−0.9–1.3	0.684
LOS in CNSM Subgroup	13.9 ± 3.2	13.8 ± 3.1	−1.2–1.4	0.812
LOS in RANSM Subgroup	14.8 ± 4.1	14.5 ± 3.9	−1.4–2.0	0.597
Drain Removal (days)	13.5 ± 3.6	13.2 ± 3.3	−0.8–1.4	0.541

Values are presented as mean ± SD (minutes). * Statistically significant (*p* < 0.05). ** Statistically significant (*p* < 0.01). ‘Total Operative Time’ includes mastectomy and reconstruction. ‘Plastic Surgery Time’ refers specifically to the reconstruction phase, representing the primary efficiency metric.

**Table 3:** Postoperative complications and aesthetic outcomes.

Variable	Tear-Drop Group (n = 155)	PMC Group (n = 72)	*p*-Value
Overall Complications	26 (16.8%)	10 (13.9%)	0.582
Major Complications (CD ≥ III)	4 (2.6%)	2 (2.8%)	0.999 ^
Implant Loss	2 (1.3%)	1 (1.4%)	0.999 ^
Skin Necrosis (Full Thickness)	2 (1.3%)	1 (1.4%)	0.999 ^
Minor Complications (CD I–II)	22 (14.2%)	8 (11.1%)	0.518
Seroma (Requiring Aspiration)	10 (6.5%)	4 (5.6%)	0.784 ^
Skin Necrosis (Partial/Linear)	8 (5.2%)	3 (4.2%)	0.999 ^
Infection (Oral Antibiotics)	3 (1.9%)	1 (1.4%)	0.999 ^
Red Breast Syndrome	1 (0.6%)	0 (0.0%)	0.999 ^
Aesthetic Outcomes			
Rippling (Palpable)	21 (13.5%)	3 (4.2%)	0.032 *

Values are presented as number (%). CD, Clavien–Dindo classification. ^ Fisher’s exact test. * Statistically significant (*p* < 0.05).

**Table 4:** BREAST-Q Scores at 6-month follow-up.

Scale (0–100)	Tear-Drop Group (n = 155)	PMC Group (n = 72)	Diff.	95% CI	*p*-Value
Satisfaction with Breasts	72.5 ± 14.1	79.8 ± 12.4	+7.3	3.1–11.5	0.001 *
Psychosocial Well-being	75.8 ± 15.2	78.2 ± 14.8	+2.4	−1.9–6.7	0.265
Sexual Well-being	64.2 ± 19.5	67.5 ± 18.2	+3.3	−2.1–8.7	0.218
Physical Well-being (Chest)	86.9 ± 10.5	90.4 ± 8.1	+3.5	0.8–6.2	0.012 *

Values are presented as mean ± SD. BREAST-Q scores range from 0 to 100 (higher = better). * *p* < 0.05. Statistical significance was determined using the independent *t*-test.

**Table 5:** Aesthetic Item Scale (AIS) scores at 6-month follow-up.

AIS Item	Tear-Drop Group (n = 155)	PMC Group (n = 72)	Mean Diff.	95% CI	*p*-Value
Breast Volume Adequacy	4.12 ± 0.62	4.18 ± 0.61	+0.06	−0.12–0.24	0.274
Breast Shape Naturalness	3.86 ± 0.82	4.21 ± 0.58	+0.35	0.14–0.56	0.001 *
Bilateral Symmetry	3.78 ± 0.88	3.95 ± 0.74	+0.17	−0.06–0.40	0.148
Contour Smoothness (Rippling)	3.71 ± 0.79	4.26 ± 0.52	+0.55	0.35–0.75	<0.001 *
Nipple–Areolar Complex	4.02 ± 0.64	4.10 ± 0.58	+0.08	−0.10–0.26	0.382
Total AIS Score	19.49 ± 3.25	20.70 ± 2.44	+1.21	0.48–1.94	0.001 *

Values are presented as mean ± SD. AIS scores range from 1 to 5 per item (higher = better). Total AIS score range: 5–25. Assessed by five blinded board-certified plastic surgeons. * *p* < 0.05.

## Data Availability

The data presented in this study are available on request from the corresponding author. The data are not publicly available due to privacy restrictions and ethical considerations regarding patient information.
